# Trait Anxiety Attenuates Response Inhibition: Evidence From an ERP Study Using the Go/NoGo Task

**DOI:** 10.3389/fnbeh.2020.00028

**Published:** 2020-03-10

**Authors:** Lisheng Xia, Licheng Mo, Jian Wang, Weifeng Zhang, Dandan Zhang

**Affiliations:** ^1^School of Psychology, Guizhou Normal University, Guiyang, China; ^2^School of Psychology, Shenzhen University, Shenzhen, China; ^3^School of Electronics and Communication, Shenzhen Institute of Information Technology, Shenzhen, China; ^4^Shenzhen Institute of Neuroscience, Shenzhen, China

**Keywords:** response inhibition, trait anxiety, event-related potential, Go/NoGo, N2, P3

## Abstract

Neuropsychology and cognitive neuroscience have shown that anxious individuals have deficits in response inhibition. However, existing knowledge about the influence of trait anxiety on response inhibition is still inconsistent. The aim of this study was to investigate response inhibition between groups with high trait anxiety (HTA) and low trait anxiety (LTA). Here, we used event-related potential (ERP) indexes as biomarkers to examine the effect of trait anxiety on response inhibition using the Go/NoGo task. Behavioral results indicated that the HTA group made significantly lower accuracy than did the LTA group in the NoGo condition but not the Go condition. Meanwhile, the HTA group needed significantly longer overall response time (RT) than the LTA group did. ERP analyses revealed that the HTA group had smaller and later frontal NoGo-N2 as well as larger and later parietal NoGo-P3 compared to the LTA group. The two response inhibition-related ERP components are distinct neurophysiological indexes that, first, the NoGo-N2 is a component involved in the motor plan prior to the motor execution inhibitory process. Second, the NoGo-P3 reflects later monitoring and evaluation of the inhibition process. Accordingly, the current ERP findings suggest that HTA individuals’ response inhibition deficits are the consequence of abnormal premotor inhibition control and inefficient evaluation and monitoring. In addition, we also found that the peak amplitude of NoGo-N2 and NoGo-P3 were significantly correlated with the State–Trait Anxiety Inventory (STAI) scores after correction for multiple comparisons. To sum up, these results support the notion that trait anxious individuals have response inhibition deficits in the Go/NoGo task.

## Introduction

According to Eysenck’s attentional control theory (Eysenck et al., 2007), anxiety might be associated with dysfunction of inhibitory control. From this perspective, neuropsychology and cognitive neuroscience studies have revealed that the medial prefrontal regions [including the anterior cingulate cortex (ACC)] are crucial substrates of the human anxiety circuitry (Sehlmeyer et al., 2009) and that deficits in these areas are associated with impaired inhibition control (Sehlmeyer et al., 2010). Although previous studies have shown that individuals with state anxiety (e.g., experimentally induced anxiety; see Aylward et al., 2017), anxiety with substance use (Karch et al., 2007), and clinical anxious patients (Grillon et al., 2017) have deficits in response inhibition, the question remains on whether and at what extent anxiety-related personality traits (e.g., trait anxiety) can also modulate response inhibition. Recent studies have demonstrated that trait anxiety interrupts top-down goal-driven processes such as response inhibition, resulting in failures in the inhibition function that enable executive control over prepotent motor responses (Pacheco-Unguetti et al., 2012; Su-Hao et al., 2014). Thus, this abnormal inhibitory function may consequently alter the level of cognitive control as well as cognitive performance in anxious population (Sehlmeyer et al., 2010) and appear to be a promising predictive marker of trait anxiety (Grillon et al., 2017). Response inhibition is a critical executive function in accordance with situation changes in everyday life, and this function involves attention and flexibility, which are largely influenced by anxiety levels (Pacheco-Unguetti et al., 2012). Moreover, investigating the response inhibition in anxiety may deepen our understanding of comorbid anxiety symptoms including impulsivity (Jakuszkowiak-Wojten et al., 2015) and substance use disorders (Karch et al., 2007). Therefore, it is important to investigate the influence of trait anxiety on response inhibition and its associated neural mechanism, which can broaden our understanding of the inhibitory control of anxious individuals and further unravel the psychological and etiological mechanisms of anxiety.

Among many inhibition tasks such as the two-choice oddball task (Wang et al., 2011; Yuan et al., 2012, 2017; Ren et al., 2019), this study employed the Go/NoGo task due to its wide application and easy performance (Helton, 2009). In this paradigm, subjects should response fast to frequently presented “Go” targets while ignoring rare “NoGo” stimuli using motor inhibition (Aron and Poldrack, 2005). Such withholding of a prepotent response generates a prototypical index of response inhibition (Helton, 2009; Bari and Robbins, 2013). To our knowledge, previous studies have examined the influence of trait anxiety on response inhibition in the Go/NoGo task and resulted in conflict results. For example, Sehlmeyer et al. (2010) found that trait anxious subjects maintain a high level of cognitive control effort reflected by electrophysiological measurements which might facilitate response inhibition. Some researchers explained that trait anxious individuals are cautious about errors and aware of their cognitive control failures. Thus, they might allocate excessive cognitive resources in response inhibition task (McWilliams and Cox, 2001; Righi et al., 2009; Sehlmeyer et al., 2010). However, some other studies found that attenuated response inhibition in trait anxiety due to the dysfunction in the frontal cortex (Yang and Li, 2014) and cognitive control deficits in anxiety resulted in impaired response inhibition (Pacheco-Unguetti et al., 2012). These results suggested that trait anxiety might attenuate rather than facilitate the response inhibition. Consistent with this idea, recent studies suggested that trait anxiety interferes with the top-down mechanisms required for the suppression of prepotent responses, resulting in failures in the response inhibition (Ansari and Derakshan, 2010, 2011). Furthermore, this abnormal response inhibition process might consequently reduce the level of cognitive control to prepare and to evaluate the outcome of actions reflected by electrophysiological measurements in trait anxious population (Yang and Li, 2014). In our opinion, the declining level of cognitive control supports the assumption that impairment of inhibitory control leads to reduced neural processing efficiency related to cognitive control in trait anxious individuals. Although the neural processing efficiency related to cognitive control in response inhibition account does not make predictions with respect to the effects of trait anxiety on behavioral performance (Basten et al., 2011), several studies proposed that less flexibility in response control in trait anxious individuals is driven by their repetitive compulsive behaviors which might induce an inability to inhibit prepotent responses reflected by behavioral performance measurements, for example, high-anxiety individuals may show an inefficient or inflexible response style with repetitive movements with rigid routines (Bannon et al., 2002; Martial et al., 2005). Thus, we assume that trait anxiety might attenuate the response inhibition that shifts motor action tendencies, resulting in cognitive failures. However, the detailed underlying mechanisms of trait anxiety modulate response inhibition related to cognitive control deficiency are far less elucidated (e.g., as mentioned above, the different patterns of electrophysiological activity related to cognitive control level in trait anxiety were not predictive of overt behavioral performance during response inhibition) and should be taken into consideration in our study.

The goal of the present study was to verify whether and at what extent the attenuated response inhibition processes in trait anxious individuals due to the impairment of cognitive control processes as shown on the electrophysiological level can also be demonstrated on the behavioral level, so as to enrich the understanding of the influence of trait anxiety on response inhibition. To this end, we chose the event-related potential (ERP) technique for its exquisite temporal resolution (Amodio et al., 2014). Two frontocentral ERP components have been associated with different subprocesses of response inhibition in the Go/NoGo task (Beste et al., 2010), based on which we compared the ERP differences between individuals with high and low trait anxiety (HTA and LTA, respectively). The first component is the frontal–midline N2, peaking approximately from 200 ms to 400 ms post stimulus. The N2 displays larger amplitudes in the NoGo compared to Go conditions (Eimer, 1993). In general, the N2 enhancement for NoGo stimuli (NoGo-N2) has been interpreted as a premotor inhibitory process that suppresses the incorrect response prior to reaction stage (Falkenstein et al., 1999). The latency of NoGo-N2 reflects the success or failure of inhibitory control (Roche et al., 2005). The amplitudes of NoGo-N2 have been found to be negatively correlated with psychiatric symptoms such as obsessive–compulsive disorder (Herrmann et al., 2003; Kim et al., 2007), depression (Katz et al., 2010), and attention-deficit/hyperactivity disorder (Woltering et al., 2013); also, a high false alarm rate (the number of mistaken responses made on NoGo trials) is associated with small and delayed NoGo-N2 (Falkenstein et al., 1999). The second component is the parietal P3, peaking approximately from 300 ms to 600 ms post-stimulus, which also displays larger amplitudes in the NoGo compared to Go conditions (Falkenstein et al., 1999). The P3 enhancement to NoGo stimuli (NoGo-P3) has been considered as an extra enhanced cognitive control effort for later monitoring and evaluation of the outcome of inhibition process (Schmajuk et al., 2006; Huster et al., 2013). In addition, the prolonged NoGo-P3 latency might reflect the extent of evaluation processing (Roche et al., 2005). Taken together, the superior response inhibition in subjects was characterized by larger and shorter NoGo-N2 as well as smaller and shorter NoGo-P3 (Zhang et al., 2015).

Based on current knowledge in the Go/NoGo task, several studies found that the attenuated response inhibition in anxious population was due to decreased activation of the frontal area and that the hypoactivity of the frontal cortex might lower premotor inhibition but enhance the cognitive control effort for monitoring and evaluation of inhibition outcomes (Kim et al., 2007; Yang and Li, 2014). The latter two processes are reflected by the NoGo-N2 and NoGo-P3. Regarding this, we predicted in this study the following. On the behavioral level, the HTA group would exhibit lower accuracy (ACC) in the NoGo condition compared with the LTA group; on the electrophysiological level, the HTA group would show smaller and later NoGo-N2 as well as larger and later NoGo-P3 compare with the LTA group.

## Materials and Methods

### Participants

In view of the fact that anxiety and depression are highly comorbid (Hirsh and Inzlicht, 2008; Nelson et al., 2016) and depressive individuals also have inhibitory deficits in the Go/NoGo task (Kaiser et al., 2003; Ruchsow et al., 2008), we only recruited nondepressed participants with HTA vs. LTA in this study.

All the freshman students (*n* = 6,903) in Shenzhen University were required to complete the Trait form of Spielberger’s State–Trait Anxiety Inventory (STAI-T; Spielberger et al., 1983; Shek, 1993). Among them, 788 questionnaires were missed. As a result, the total effective sample of questionnaires was 6,115, and the effective rate was 88.6%. In this sample, individuals with STAI-T scores in the upper and lower 25% of the distribution were considered as HTA and LTA subjects (Gu et al., 2010; Luo et al., 2014; Xia et al., 2017). The Beck Depression Inventory second edition (BDI-II; Beck et al., 1996) was used to assess self-reported symptoms of depression. Only the participants with BDI-II scores <13 were considered in this study (note that while BDI-II < 13 indicates minimal depression, BDI-II ≥ 14 indicates mild, moderate, or severe depression; see Beck et al., 1996). From those who met these criteria, we randomly recruited 56 students as paid participants (28 in the LTA group and 28 in the HTA group). There was no significant difference between the two groups with respect to age, handedness, and BDI-II scores (see Table 1).

**Table 1:** Demographic data of participants with high trait anxiety (HTA) and low trait anxiety (LTA).

Characteristics	LTA (*n* = 28)	HTA (*n* = 28)	Statistics
Mean age, years	20.10 ± 1.13	19.80 ± 1.02	*t*_(54)_ = 1.01, *p* = 0.153
Sex, male/female	14/14	14/14	
Handedness, right/left	28/0	28/0
STAI-T	31.79 ± 5.43	55.68 ± 4.30	*t*_(54)_ = −17.928, *p* < 0.001
BDI-II	4.32 ± 1.42	4.96 ± 1.59	*t*_(54)_ = −1.568, *p* = 0.123

*STAI-T, the trait form of Spielberger’s State–Trait Anxiety Inventory; BDI-II, Beck Depression Inventory (second edition). Descriptive data are presented as mean ± standard deviation*.

Exclusion criteria for both groups were: (1) any Axis I and II disorders according to the Diagnostic and Statistical Manual (DSM-IV; APA, 1994); (2) seizure disorder; (3) history of head injury with possible neurological sequelae; and (4) substance abuse or dependence in the past 6 months.

### Procedures

**Figure 1:**
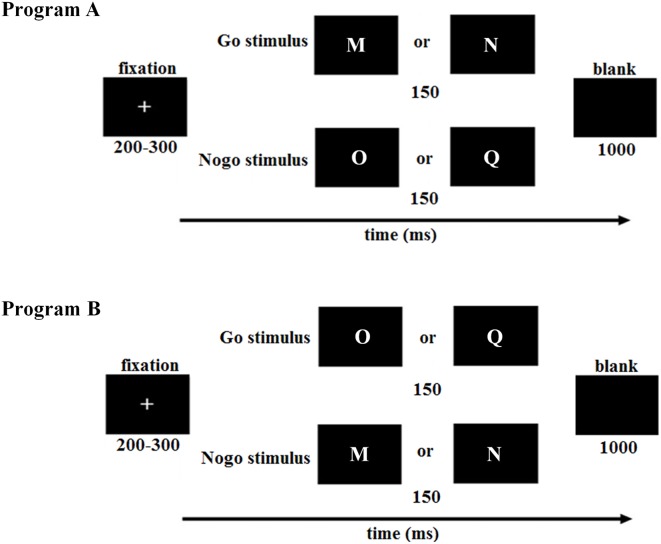
Illustration of the Go/NoGo paradigm in this study. To counterbalance the Go and NoGo stimuli across subjects, the first half of low trait anxiety (LTA) and high trait anxiety (HTA) subjects was assigned to program **(A)**, while the second half was assigned to program **(B)**. LTA, the low-trait anxiety group; HTA, the high-trait anxiety group.

Each trial started with a 200- to 300-ms fixation, followed by targets (Go stimuli: M, N or O, Q) and nontargets (NoGo stimuli: O, Q or M, N) that were presented for 150 ms (see also Kim et al., 2007). A black blank screen appeared after the stimulus and lasted for 1,000 ms. Participants were required to press a button with their right index finger on the response box as quick as possible when the Go stimuli appeared while with hold the motor responses when the NoGo stimuli appeared. The Go and NoGo trials were presented in random order with a probability of 2:1 to build a prepotent response of “Go” (see also Yang et al., 2010). The total experiment consisted of two blocks, with 240 trials in each block (Go stimulus: 160 trials; NoGo stimulus: 80 trials). In order to avoid the confounding factor of letter shape, the Go and NoGo stimuli were counterbalanced across subjects (Figure 1).

### EEG Recording and Analysis

Brain electrical activity was recorded referentially against the left mastoid and re-referenced off-line to the average of the left and right mastoids, by a 64-channel amplifier with a sampling frequency of 250 Hz (Brain Products, Gilching, Germany). Electroencephalography (EEG) data were collected with electrode impedances kept below 5 kΩ. Ocular artifacts were removed from EEGs using a regression procedure implemented in Neuroscan software (Scan 4.3).

The recorded EEG data were filtered (0.01−30 Hz; slope 12 dB/oct; zero phase) and segmented beginning 200 ms prior to the onset of stimuli. All epochs were baseline-corrected with respect to the mean voltage over the 200 ms preceding the onset of stimuli, followed by averaging in association with Go and NoGo conditions. Trials contaminated with large artifacts (peak-to-peak deflection exceeded ±100 μV) were excluded from the averaging. As a result, 21 ± 8 trials and 13 ± 7 trials were rejected in each subject for Go and NoGo conditions, respectively. The rejected trials were less than 10% of the total trials (see also Gu et al., 2010; Xia et al., 2017). Trial numbers did not show significant difference between experimental conditions.

We analyzed the peak amplitudes and peak latencies of the frontal–midline N2 and parietal P3; the measures were averaged based on waveforms of different sets of electrodes according to grand-mean ERP topographies and relevant literatures (Kim et al., 2007; Huang et al., 2009; Righi et al., 2009). The N2 peak was detected to occur at 250–350 ms post stimuli at the electrode sites of Fz, F1, F2, FCz, FC1, FC2, Cz, C1, and C2, while the P3 peak was detected within a time window of 340–420 ms (Go condition) or 430–530 ms (NoGo condition) at the electrode sites of Pz, P1, P2, CPz, CP1, CP2, and POz.

### Statistics

Statistical analysis was performed using SPSS Statistics 21.0 (IBM, Somers, NY, USA). Descriptive data were presented as mean ± standard error. The significance level was set at 0.05.

Two-way repeated-measures ANOVAs were performed on measurements of behavioral [ACC and response time (RT)] and ERP data (N2 and P3 amplitude/latency), with response assignment (Go vs. NoGo) as the within-subject factor and group (HTA vs. LTA) as the between-subject factor. Significant interactions were analyzed using simple effects model. Partial eta-squared ($\eta_{\text{p}}^{2}$) was reported to demonstrate the effect size in ANOVA tests.

Two-tailed Pearson’s *r* correlation was performed between the two self-reported measures (BDI-II and STAI-T) and behavioral/ERP indexes. Correction for multiple comparisons was based on Holm’s stepwise method.

## Results

For the sake of brevity, the experimental effects that did not reach significance were omitted.

### Behaviors

#### Accuracy

**Figure 2:**
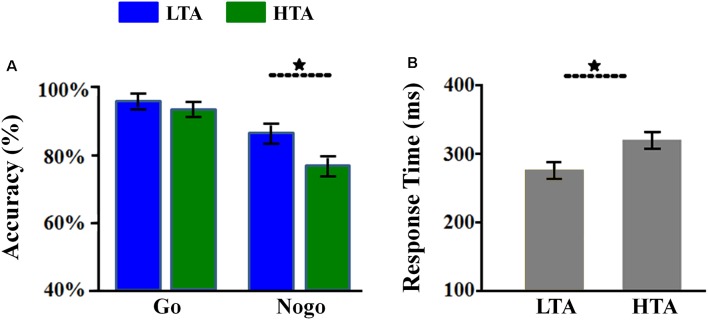
Behavioral results. **(A)** The Go accuracy and NoGo accuracy in the two groups. **(B)** The overall response time (RT) in the two groups. Bars represent standard error of the mean. LTA, the low-trait anxiety group; HTA, the high-trait anxiety group. *p < 0.05.

The interaction of response assignment by group was significant (*F*_(1,54)_ = 4.107; *p* = 0.048; $\eta_{\text{p}}^{2}$ = 0.071; Figure 2A). Simple effect analysis indicated that the ACC in the NoGo trials was lower in the HTA group (76.05 ± 2.96%) compared with that in the LTA group (86.75 ± 2.96%; *F*_(1,54)_ = 6.525, *p* = 0.013). However, this group difference did not achieve significance level in the Go trials (*F*_(1,54)_ < 1; HTA = 93.07 ± 2.06% ; LTA = 95.78 ± 2.06%).

The main effect of group was significant (*F*_(1,54)_ = 4.926; *p* = 0.031; $\eta_{\text{p}}^{2}$ = 0.084). The HTA group (84.56 ± 2.14%) made lower ACC than the LTA group did (91.26 ± 2.14%).

#### Response Time

The main effect of group was significant (*F*_(1,54)_ = 7.733; *p* = 0.007; $\eta_{\text{p}}^{2}$ = 0.125; Figure 2B). The LTA group (overall RT: 274.77 ± 10.93 ms; Go RT for correct response: 295.82 ± 14.36 ms) responded much faster than the HTA group did (overall RT: 317.75 ± 10.93 ms; Go RT for correct response: 322.25 ± 14.36 ms).

### ERPs

#### N2

**Figure 3:**
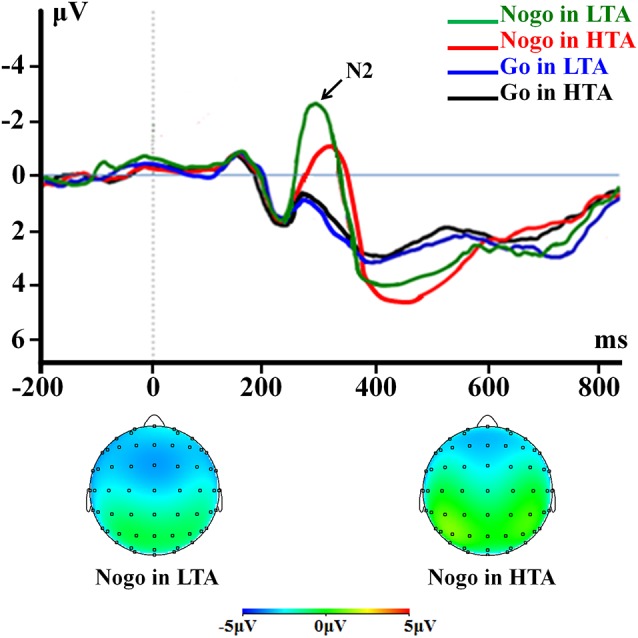
The N2 component time-locked to the Go and NoGo conditions. Event-related potentials (ERPs) were calculated by averaging the data at the electrodes of Fz, F1, F2, FCz, FC1, FC2, Cz, C1, and C2.

For peak amplitude, the interaction effect of response assignment by group was significant (*F*_(1,54)_ = 4.418; *p* = 0.040; $\eta_{\text{p}}^{2}$ = 0.076; Figure 3). Simple effect analysis indicated that the N2 amplitude in the NoGo condition (*F*_(1,54)_ = 9.567; *p* = 0.003) was lower in the HTA group (−1.35 ± 0.31 μV) compared with the LTA group (−2.71 ± 0.31 μV). However, this group difference did not achieve significant level in the Go condition (*F*_(1,54)_ < 1; HTA = 0.76 ± 0.28 μV; LTA = 0.77 ± 0.28 μV). The main effect of group was significant (*F*_(1,54)_ = 6.567; *p* = 0.013; $\eta_{\text{p}}^{2}$ = 0.108). The HTA group (−0.29 ± 0.19 μV) had a smaller N2 than the LTA group did (−0.97 ± 0.19 μV).

For peak latency, the interaction effect of response assignment by group was significant (*F*_(1,54)_ = 7.946; *p* = 0.007; $\eta_{\text{p}}^{2}$ = 0.128; Figure 3). Simple effect analysis indicated that the N2 latency in the NoGo condition (*F*_(1,54)_ = 11.475; *p* = 0.001) was longer in the HTA group (336.71 ± 8.08 ms) compared with LTA group (298.00 ± 8.08 ms), However, this group difference did not achieve significance level in the Go condition (*F*_(1,54)_ < 1; HTA = 264.50 ± 7.12 ms; LTA = 268.75 ± 7.12 ms). The main effect of group was significant (*F*_(1,54)_ = 5.121; *p* = 0.028; $\eta_{\text{p}}^{2}$ = 0.087). The HTA group (300.61 ± 5.38 ms) had a longer peak latency of the average N2 than the LTA group did (283.38 ± 5.38 ms).

#### P3

**Figure 4:**
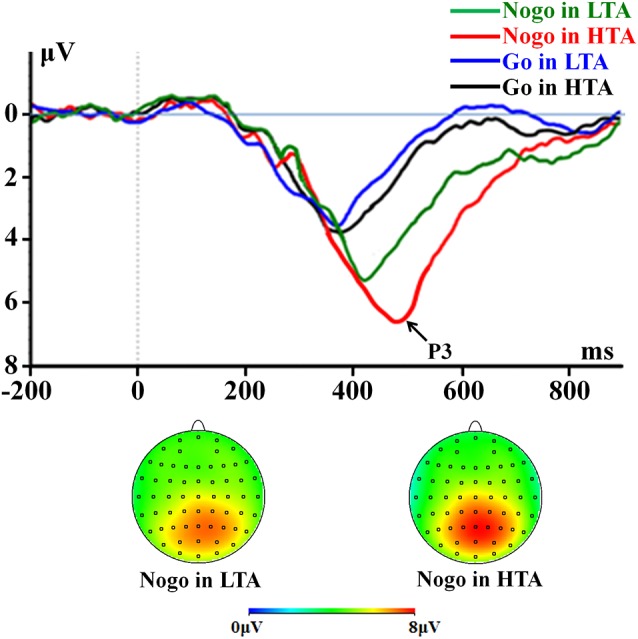
The P3 component time-locked to the Go and NoGo conditions. ERPs were calculated by averaging the data at the electrodes of Pz, P1, P2, CPz, CP1, CP2, and POz.

For peak amplitude, the interaction effect of response assignment by group was significant (*F*_(1,54)_ = 4.534; *p* = 0.038; $\eta_{\text{p}}^{2}$ = 0.077; Figure 4). Simple effect analysis indicated that the P3 amplitude in the NoGo condition (*F*_(1,54)_ = 11.496; *p* = 0.001) was higher in the HTA group (6.63 ± 0.33 μV) compared with the LTA group (5.04 ± 0.33 μV). However, this group difference did not achieve significance level in the Go condition (*F*_(1,54)_ < 1; HTA = 3.55 ± 0.33 μV; LTA = 3.37 ± 0.33 μV). The main effect of group was significant (*F*_(1,54)_ = 7.125; *p* = 0.01; $\eta_{\text{p}}^{2}$ = 0.117). The HTA group (5.09 ± 0.24 μV) had a larger P3 than the LTA group did (4.20 ± 0.24 μV).

For peak latency, the interaction effect of response assignment by group was significant (*F*_(1,54)_ = 6.603; *p* = 0.013; $\eta_{\text{p}}^{2}$ = 0.109; Figure 4). Simple effect analysis indicated that the P3 latency in the NoGo condition (*F*_(1,54)_ = 16.664; *p* < 0.001) was longer in the HTA group (473.86 ± 11.82 ms) compared with LTA group (405.64 ± 11.82 ms). However, this group difference did not achieve significance level in the Go condition (*F*_(1,54)_ < 1; HTA = 376.75 ± 11.17 ms ; LTA = 372.93 ± 11.17 ms). The main effect of group was significant (*F*_(1,54)_ = 12.076; *p* = 0.001; $\eta_{\text{p}}^{2}$ = 0.183). The HTA group (425.30 ± 7.33 ms) had a longer peak latency of the average P3 than the LTA group did (389.29 ± 7.33 ms).

#### Correlation

According to the results reported above, we conducted Pearson correlation analyses between the two self-reported scores (STAI-T and BDI) and the five behavioral/ERP indexes which showed interaction between response assignment and group (e.g., the ACC in the NoGo condition and the peak amplitudes as well as peak latencies of NoGo-N2 and NoGo-P3). In total, we performed 10 (2 × 5) correlations.

The results showed two significant correlations after correction for multiple comparisons. The peak amplitudes of NoGo-N2 (*r* = 0.383, *p* = 0.004, corrected *p* = 0.036; note that since NoGo-N2 is a negative-going component, the positive correlation means that higher STAI-T scores were associated with smaller NoGo-N2 amplitudes) and NoGo-P3 were correlated with the STAI-T (*r* = 0.404, *p* = 0.002, corrected *p* = 0.02).

In addition, we also conducted Pearson correlation analyses between the behavior and ERP which showed interaction between response assignment and group (e.g., the ACC in the NoGo condition and the peak amplitudes as well as peak latencies of NoGo-N2 and NoGo-P3). In total, we performed 4 (1 × 4) correlations.

The results showed one significant correlation after correction for multiple comparisons. The peak amplitudes of NoGo-N2 were correlated with ACC (*r* = −0.409, *p* = 0.002, corrected *p* = 0.02).

## Discussion

Debate exists on the influence of trait anxiety on response inhibition. This study applied the Go/NoGo paradigm to compare the response inhibition between HTA and LTA participants. On the behavioral level, we found that the HTA group made significantly lower ACC than the LTA group did in the NoGo condition. Meanwhile, the overall RT was longer in the HTA group than in the LTA group. Our behavioral results are consistent with the study conducted by Pacheco-Unguetti et al. (2012) that showed impaired response inhibition processes due to inflexibility in response control in anxiety. However, our behavioral results are inconsistent with some other studies showing no behavioral effect of trait anxiety in the Go/NoGo paradigm (Karch et al., 2007; Ruchsow et al., 2008; Righi et al., 2009). These discrepancies may be due to the difference in experimental parameters. For example, the study conducted by Righi et al. (2009) required subjects to inhibit their response to only one special stimulus, and all stimuli were presented for 250 ms. However, in this study, subjects were required to discriminate between two pairs of stimuli, and these stimuli were presented for 150 ms, which enhanced the difficulty of inhibiting the NoGo stimuli. In addition, the ratio between Go and NoGo trials may modulate the behavioral results (Kim et al., 2007). In our study, we used a 2:1 Go/NoGo ratio while Karch et al. (2007) and Ruchsow et al. (2008) used a 1:1 ratio.

As mentioned in the “Introduction” section, we speculate that the attenuated response inhibition behavioral effect in trait anxious individuals is driven by their repetitive compulsive behaviors. The core symptoms of repetitive compulsive behaviors (e.g., rigid routines, hesitation, and inflexibility) have been thought to be related to response inhibition deficits (Bannon et al., 2002), and this deficit might serve as a behavioral maker underlying inhibitory dysfunction of anxiety findings (Martial et al., 2005; Kim et al., 2007). Moreover, several evidences suggested that repetitive compulsive behaviors and trait anxiety are highly positive related (Black, 2008; Rodgers et al., 2012; Goodwin, 2015). It has been found that HTA individuals showed an inability to inhibit certain stimuli or certain prepotent responses compared with LTA individuals due to the repetitive compulsive behaviors (Martial et al., 2005). Meanwhile, HTA participants are less flexible in response control (e.g., inability to set shift) than the LTA group due to repetitive compulsive behavior inducing slowness and hesitation (Hughes et al., 2008; Dar and Iqbal, 2014; Yilmaz, 2015), resulting in a decrease in speed in the task. Note that this study also found that the Go RT was slightly longer in the HTA group than in the LTA group. In the current study, both the ACC and the RT indicate that HTA individuals are inferior to LTA individuals in response inhibition, leading to decreased NoGo ACC and longer RT. However, a non-negligible limitation of the current study is that we did not include any behavioral measure of the repetitive compulsive level, such as the Yale-Brown Obsessive Compulsive Scale (Goodman et al., 1989) and Repetitive Behaviors Scale (Lam and Aman, 2007). Follow-up research is necessary to further address this issue.

On the electrophysiological level, the first ERP finding is that trait anxious participants showed smaller and later NoGo-N2 compared to low-anxiety participants. Consistent with this result, previous Go/NoGo studies found that a smaller and/or later NoGo-N2 was evoked in individuals with obsessive–compulsive disorder (Herrmann et al., 2003; Kim et al., 2007), depression (Katz et al., 2010), and attention-deficit/hyperactivity disorder (Woltering et al., 2013). As mentioned in the introduction, the NoGo-N2 reflects the inhibitory process of a motor plan prior to the motor execution stage (Eimer, 1993; Falkenstein et al., 1999; Herrmann et al., 2003; Karch et al., 2007; Kim et al., 2007; Huster et al., 2013), and this inhibitory process is usually located at the premotor level rather than at the motor level (Falkenstein et al., 1999). The N2 enhancement to the NoGo stimulus has been suggested to reflect the suppression of incorrect response prior to the motor action (Falkenstein et al., 1999; Zhang et al., 2015), and a shorter-latency NoGo-N2 has been observed in successful withholding to NoGo stimuli compared with unsuccessful attempts to inhibit (Roche et al., 2005). In addition, neuroimaging studies have revealed that the neural sources of NoGo-N2 are located in the ACC and inferior/orbitofrontal prefrontal cortex (Kiefer et al., 1998; Bokura et al., 2001; Bekker et al., 2005). In our opinion, the smaller and later NoGo-N2 in the frontocentral electrode sites observed in anxious individuals here indicates that trait anxiety is associated with dysfunction in the frontal prefrontal cortex (including the ACC), which are crucial neural substrates known to be involved with the anxiety circuitry (Sehlmeyer et al., 2010). Taken together, the current finding of anxiety-modulated NoGo-N2 suggests that the premotor inhibitory process of trait anxious individuals is impaired, which might disrupt behavioral responses and inhibition.

The second ERP finding is that trait anxious participants were associated with an enhanced and latency-prolonged NoGo-P3 component. The NoGo-P3 is usually considered as the later monitoring and evaluation of the inhibition process outcome (Beste et al., 2010; Huster et al., 2013). The P3 enhancement to NoGo stimuli has been considered as more effort being devoted to monitoring of behavioral outcome (Sehlmeyer et al., 2010), while the prolonged NoGo-P3 latency is thought to be indicative of deliberative or “deeper” evaluation (Roche et al., 2005). Several studies related to response inhibition revealed that anxious individuals need to allocate more cognitive resources and make more control effort to withhold a response compared with healthy individuals, leading to a larger and/or longer NoGo-P3 (Karch et al., 2007; Ruchsow et al., 2007). Consistent with this idea, the current finding shows that the NoGo stimulus evoked larger and later NoGo-P3 in anxious individuals, suggesting that anxiety impairs inhibitory control system and makes us unable to evaluate and monitor the inhibition of incorrect responses in an efficient manner, leading to enhanced cognitive control effort or extra processing resources in the brain of anxious people.

Finally, one limitation should be pointed out for an appropriate interpretation of the current result. This study only measured the response inhibition in young, anxious adults (approximately 20 years old). Seeing that the cognitive characteristics of anxious people might differ between the young-adult group and other age groups (Krasucki et al., 1998; Goldberg et al., 2003), the generalizability of the current findings awaits to be tested in future work.

To sum up, this study has revealed that HTA participants have response inhibition deficits in the Go/NoGo paradigm, demonstrating a negative relationship between trait anxiety and response inhibition. The ERP results indicate that the psychological processes of premotor (reflected by NoGo-N2) and later evaluation of inhibition processes (reflected by NoGo-P3) both contribute to impaired response inhibition in anxiety. Specifically, HTA individuals’ response inhibition deficits are due to deficits of premotor inhibition control and inefficient evaluation and monitoring of NoGo stimuli. These findings would provide valuable knowledge about the underlying mechanism of the maladaptive response inhibition in trait anxious people.

## Data Availability Statement

All datasets generated for this study are included in the article.

## Ethics Statement

The studies involving human participants were reviewed and approved by the Ethics Committee of Institute of Psychology, Chinese Academy of Sciences (H14019). The patients/participants provided their written informed consent to participate in this study.

## Author Contributions

LX and DZ designed the study. LX conducted the experiment and analyzed the data. LX, LM, JW, WZ, and DZ contributed to the manuscript.

## Conflict of Interest

The authors declare that the research was conducted in the absence of any commercial or financial relationships that could be construed as a potential conflict of interest.
